# Three Chitin Deacetylase Family Members of Beauveria bassiana Modulate Asexual Reproduction and Virulence of Fungi by Mediating Chitin Metabolism and Affect Fungal Parasitism and Saprophytic Life

**DOI:** 10.1128/spectrum.04748-22

**Published:** 2023-02-14

**Authors:** Jia-Hua Liu, Jing-Chong Dong, Jun-Jie Gao, Xin-Peng Li, Shun-Juan Hu, Juan Li, Wen-Xiao Hu, Xian-Yan Zhao, Juan-Juan Wang, Lei Qiu

**Affiliations:** a State Key Laboratory of Biobased Material and Green Papermaking, Qilu University of Technology, Shandong Academy of Sciences, Jinan, China; b School of Biological Science and Technology, University of Jinan, Jinan, China; The Ohio State University

**Keywords:** chitin deacetylase, entomopathogenic fungi, virulence, asexual growth

## Abstract

As an important chitin-modifying enzyme, chitin deacetylase (CDA) has been characterized in many fungi, but its function in the entomopathogenic fungus Beauveria bassiana remains unclear. Three CDAs with conserved domains of the carbohydrate esterase 4 (CE-4) family were identified in B. bassiana. Disruption of *CDA1* resulted in growth restriction of the fungus on medium with chitin as a carbon source or without a carbon source. Deletion of *CDA1* and *CDA2* led to defects in fungal conidial formation and conidial vitality compared with those of the wild type (WT), and the conidial yield decreased by 25.81% to 47.68%. Inactivation of three *CDA* genes resulted in a decrease of 20.23% to 27% in the blastospore yield. Δ*CDA1* and Δ*CDA3* showed 29.33% and 23.34% reductions in cuticular infection virulence, respectively. However, the CDA family may not contribute to hemocoel infection virulence. Additionally, the sporulation of the insect carcass showed that the three gene deletion mutants were 68.45%, 63.84%, and 56.65% less than WT. Penetration experiments with cicada wings and enzyme activity assays were used to further explore the effect of the fungus on chitin metabolism after gene deletion. Although the three gene deletion mutants penetrated the cicada wings successfully and continued to grow on the underlying medium, their colony sizes were reduced by 29.12% to 47.76%. The CDA enzyme activity of Δ*CDA1* and Δ*CDA3* decreased by 84.76% and 83.04%, respectively. These data showed that members of the CDA family play a different role in fungal growth, conidial quality, and virulence.

**IMPORTANCE** In this study, we report the roles of CDA family in entomopathogenic fungus B. bassiana. Our results indicated that CDA modulates asexual development and regulates fungal virulence by altering chitin deacetylation and metabolic capacity. CDA affected the biological control potential and life history of B. bassiana by affecting its parasitic and saprophytic life. These findings provide novel insights into the roles of multiple CDA paralogues existing in fungal biocontrol agents.

## INTRODUCTION

Beauveria bassiana is a fungus that causes white muscardine disease in many arthropods and has attracted great interest because of its excellent potential for application in biological pest control (1). In nature, B. bassiana occurs in two infection stages, saprophagy and parasitization. Conidia adhere to the host cuticle and germinate under appropriate conditions. The first site and barrier of fungal interaction with the host are performed by the insect cuticle and fungal cell wall (2). A series of hydrolytic enzymes, such as chitinases and proteases, which are vital for penetrating the insect epidermis, are secreted during B. bassiana infection (3). After successful penetration using enzymes and mechanical force, the fungal mycelium undergoes two-phase morphological transformation to form a yeast-like mycelium that can resist host defense and colonize the hemocoel (4). When the fungi consume host nutrients, the hyphal penetrate the hemocoel, grow saprophytically on the cadaver surface, and generate new conidia to start the next infection cycle (5).

Chitin, a homopolymer of *N-acetylglucosamine* (GlcNAc) that is β-(1/4)-linked, is a vital element of the fungal cell wall and insect exoskeleton (6–10). Chitosan, as the product of chitin catalyzed by chitin deacetylase (CDA), is a more soluble and flexible substance than chitin (6). The synthesis of chitosan in *Mucor rouxifi* requires CDA working in tandem with chitin synthetase (11). The acetyl amino group of the chitin and chitosan GlcNAc residues can be hydrolyzed by CDA to produce GlcN units and acetic acid (12). In Saccharomyces cerevisiae, two CDAs are responsible for the correct formation of ascospore walls, and Cda2p plays a major role in this process (13–15). The *cda1* mRNA content in Schizosaccharomyces pombe increased during the spore formation stage, and *cda1* was shown to be required for sporulation (16). In filamentous fungi, a certain degree of deacetylation of nascent chitin chains appears to be significant in preventing chitin from forming overly crystalline structures (11). During the whole process of vegetative development and asexual maturation, six CDAs in *Trichoderma atroviride* perform a vital role in cell wall plasticity (17). In Aspergillus nidulans, the degradation of chitin in the cell wall during autolysis may be related to CDA (18).

In Cryptococcus neoformans, CDA converts chitin in the cell wall to chitosan, which is an important determinant of pathogenicity and cell wall integrity (19, 20). The *CDA*-encoding gene *CBP1* of Magnaporthe oryzae induces the formation and differentiation of appressorium deconstruction, and chitosan is essential for germ adhesion (21, 22). The pathogenicity of Podosphaera xanthii is affected by the CDA protein, as fungal growth was inhibited and the plant defense response was dramatically stimulated after *CDA* gene silencing (23). The process of chitin deacetylation may be significant for the formation of fungal cell structures and fungus-host interactions (24).

Insect pathogenic fungi (such as Metarhizium anisopliae and B. bassiana) have emerged as suitable model systems for studying fungal growth and pathogen-host interactions (3). Genes related to chitin synthesis and metabolism in insect pathogenic fungi have been studied. For example, the seven chitin synthetase genes of Metarhizium acridum have different functions in vegetative growth, cell wall integrity, stress response, and pathogenicity (25). *M. anisopliae* CDA contributes significantly to chitin hydrolysis and might play a vital role in disease by softening the insect cuticle to help mycelial penetration (26). Several transcription factors that regulate chitin synthesis and metabolism have been characterized. For example, the Zn(II)_2_Cys_6_ transcriptional regulator BbTpc1 of *B.* bassiana affects the global transcriptome and contains a set of chitin synthetase genes to regulate chitin biosynthesis in fungi (27). Ron1, an Ndt80-like transcription factor, directly regulates the induction of *ChiV4* (a chitinase gene) and *ChsVII* (a chitin synthase gene) to participate in chitin catabolism and synthesis in B. bassiana (28).

Although the roles of CDA in plant-pathogenic fungi have been studied (17, 29), the deacetylation of chitin in the entomopathogenic fungus B. bassiana has not been reported. In this study, the potential effects of the B. bassiana *CDA* family genes on biocontrol potential were investigated through gene deletion and complementation.

## RESULTS

### CDA phylogenetic tree, protein structure prediction, and transcriptional analysis.

**FIG 1:**
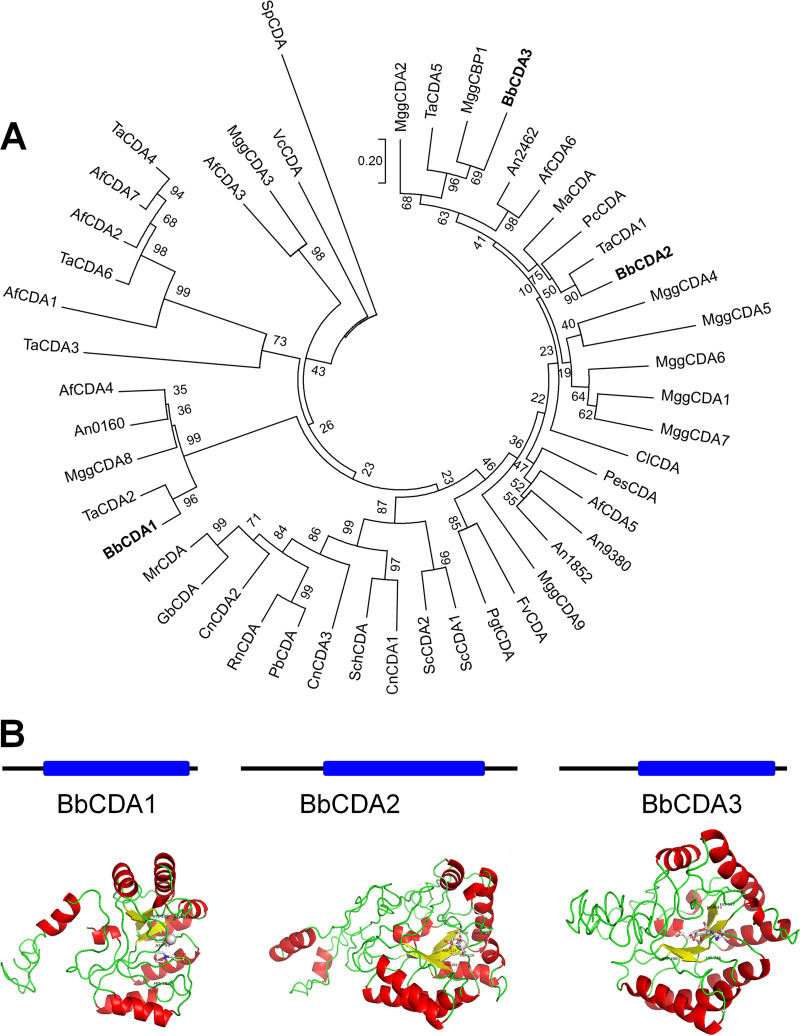
Phylogenetic and structure analyses of three identified CDAs in *B.* bassiana. (A) A phylogenetic tree was generated by using MEGA-X software. Bootstrap values are used to denote the number next to the node. The bar marker represents the genetic distance, which is proportional to the number of amino acid substitutions Complete or relevant enzyme groups for analysis of CDA were mined from the database as follows: Af, Aspergillus fumigatus; An, Aspergillus nidulans; Bb, Beauveria bassiana; Cl, *Colletotrichum lindemuthianum*; Cn, Cryptococcus neoformans; Fv, *Flammulina velutipes*; Gb, *Gongronella butleri*; Ma, Metarhizium *anisopliae*; *M*r, *Mucor rouxianus*; Mgg, *Magnaporthae oryzae*; Pb, *Phycomyces blakesleeanus*; Pc, *Pochonia chlamydosporia*; Pes, *Pestalotiopsis* sp.; Pgt, *Puccinia graminis*; Rn, *Rhizopus nigricans*; Sc, Saccharomyces cerevisiae; Sch, *Schizophyllum commune*; Sp, Schizosaccharomyces pombe; Ta, *Trichoderma atroviride*; Vc, Vibrio cholerae. (B) I-Tasser online server (https://zhanggroup.org/I-TASSER/.) was used to predict the 3D structure of CDAs. Three CDA proteins containing the NodB-like DNA-binding regions are indicated by blue lines. Secondary structure pigmented CDA structure; yellow = chain, red = helix, green = loop/coil. Side chains of metal-binding residues, catalytic acids, and catalytic bases are labeled as rods, and metal ions are shown as gray spheres.

BLAST analysis was conducted using known CDA sequences from S. cerevisiae, and three CDA protein sequences containing the NodB domain DNA-binding region were identified in B. bassiana, including CDA1 (XP_008593420.1, 248 aa), CDA2 (XP_008601714.1, 351 aa), and CDA3 (XP_008600939.1, 289 aa) (Fig. 1). CDA1 shared 21.8% and 19.7% sequence identity with CDA2 and CDA3, respectively. The sequence identity between CDA2 and CDA3 was 33.8%, indicating that CDA2 and CDA3 are closely related (Fig. 1A). The sequence relationships between the B. bassiana CDAs and S. cerevisiae CDAs, A. nidulans CDAs, M. oryzae CDAs, and T. atroviride CDAs were 17.6% to 21.3%, 18.7% to 48%, 9.3% to 53.7% and 12.6%–75.1%, respectively.

The 3D structure of the CDAs modeled by the I-TASSER server exhibited a (β/α)_8_ barrel fold (Fig. 1B). Seven (CDA3) or eight (CDA1, CDA2) parallel β-strands make up the central core of the proteins, and these β-strands form an extremely twisted β-barrel surrounded by α-helices. The predicted 3D structure of the three CDA proteins showed highly conserved His and Asp residues (Fig. 1B). CDA1 and CDA2 contain Zn^2+^ metal-binding residues, which are not found in CDA3. Consensus prediction of GO terms for the CDA domain showed that the GO-Score between the three CDAs and the template protein (GO:0005975), which is mainly involved in carbohydrate metabolism, were 0.70 to 0.89. These results suggest that CDAs may have functions similar to that of the template protein.

The putative signal peptide site was predicted by SignalP 4.1, showing that all the three CDAs were enzymes with signal peptides. The cleavage sites were located between the positions of No. 21 and 22, No. 22 and 23, or No. 23 and 24 on the N terminals of CDA1, CDA2, and CDA3, respectively (Fig. S1A to C). To assess transcriptional change of CDAs over the time of WT development during the various stages, the stages of the mycelium growth, conidiation, and blastospore production were simulated by culturing WT on SDAY plate for 4 days, on SDAY plate for 7 days and in NLB for 4 days, respectively. The quantitative real-time PCR (qRT-PCR) analysis demonstrated that the transcription levels of CDA1 and CDA3 were downregulated in conidiation stage compared with that of mycelium growth stage. In NLB, there was no significant difference in the transcription level of CDAs compared with the stage of mycelial growth (Fig. S1D). In the infection process, CDA1 and CDA3 had the highest transcript level during the early stage of infection, while the transcription level of CDA2 was upregulated on the third day of infection (Fig. S1D).

To assess the roles of CDAs in *B.* bassiana, deletion and complementation strains were created and validated by PCR and qRT-PCR (Fig. S2).

### Characterization of the role of CDAs in vegetative growth.

After 7 days of cultivation at 25°C in different media, colony areas (cm^2^) were calculated and used as an index of hyphal growth capacity. The colony sizes of Δ*CDA1*, Δ*CDA2*, and Δ*CDA3* on SDAY, 1/4 SDAY, CZA, and CZA-N were similar to the colony sizes of the control strains (Table 1, *P* > 0.05 Fig. S3). Δ*CDA2* and Δ*CDA3* showed no difference compared with the control strains (Table 1, *P* > 0.05) on CZA-C and CZA-CN. However, compared with those of WT, the colony sizes of Δ*CDA1* decreased by 12.54% and 10.62% on CZA-C and CZA-CN, respectively (Table 1, *P* < 0.05). To further explore the growth of Δ*CDA1* on different C sources, we replaced the C sources in the culture medium. Δ*CDA1* showed the same growth features as the control strains when the C source was replaced with GlcNAc. However, the colony size of Δ*CDA1* was 25% less than that of WT on medium with chitin as the C source (Table 1, *P* < 0.05). These results suggest that CDA1 may perform a vital function in the utilization of C sources, especially chitin.

**TABLE 1:** Colony sizes of *B.* bassiana strains grown on nutrition-rich and limited media for 7 days at 25°C^*a*^

Medium	Mean (± SD) colony area (cm^2^)						
	WT	Δ*CDA1*	Δ*CDA2*	Δ*CDA3*	*CDA1* ^C^	*CDA2* ^C^	*CDA3* ^C^
SDAY	3.08 ± 0.30a	2.99 ± 0.27a	3.00 ± 0.40a	3.41 ± 0.25a	3.04 ± 0.09a	2.89 ± 0.23a	3.37 ± 0.49a
1/4SDAY	2.92 ± 0.17a	2.69 ± 0.15a	2.79 ± 0.09a	2.64 ± 0.08a	2.94 ± 0.18a	3.04 ± 0.18a	2.74 ± 0.17a
CZA	2.11 ± 0.13a	2.02 ± 0.25a	2.14 ± 0.13a	2.05 ± 0.07a	2.27 ± 0.00a	2.36 ± 0.16a	2.41 ± 0.14a
C deleted	2.55 ± 0.07a	2.23 ± 0.08b	2.50 ± 0.08a	2.45 ± 0.08a	2.50 ± 0.08a	2.59 ± 0.08a	2.50 ± 0.08a
GLC as C	2.95 ± 0.15a	2.79 ± 0.31a	2.94 ± 0.18a	2.89 ± 0.09a	2.99 ± 0.27a	2.84 ± 0.00a	2.79 ± 0.09a
Chitin as C	1.92 ± 0.10a	1.43 ± 0.11c	1.81 ± 0.07ab	1.65 ± 0.11bc	1.97 ± 0.07a	1.85 ± 0.14ab	1.89 ± 0.12ab
N deleted	3.86 ± 0.15a	3.41 ± 0.25b	3.57 ± 0.10ab	3.74 ± 0.10ab	3.80 ± 0.17ab	3.80 ± 0.17ab	3.92 ± 0.10a
C&N deleted	2.07 ± 0.09a	1.85 ± 0.07b	2.05 ± 0.07ab	2.05 ± 0.07ab	2.10 ± 0.07a	2.23 ± 0.08a	2.10 ± 0.07a
NaCl	1.73 ± 0.20abc	2.18 ± 0.15a	1.65 ± 0.11bc	1.77 ± 0.00abc	2.10 ± 0.29ab	1.58 ± 0.17c	1.69 ± 0.13bc
Sorbitol	1.59 ± 0.27a	1.45 ± 0.37a	1.77 ± 0.12a	1.85 ± 0.14a	1.47 ± 0.12a	1.62 ± 0.13a	1.77 ± 0.00a
H_2_O_2_	1.90 ± 0.17a	2.05 ± 0.07a	2.10 ± 0.19a	2.05 ± 0.07a	1.93 ± 0.14a	2.10 ± 0.29a	1.77 ± 0.00a
MND	1.72 ± 0.14a	1.65 ± 0.11a	1.77 ± 0.12a	1.79 ± 0.03a	1.71 ± 0.06a	1.63 ± 0.12a	1.81 ± 0.07a
CR	1.59 ± 0.06a	1.47 ± 0.06a	1.70 ± 0.24a	1.66 ± 0.31a	1.58 ± 0.06a	1.47 ± 0.06a	1.54 ± 0.00a
SDS	1.85 ± 0.09a	1.93 ± 0.07a	2.04 ± 0.25a	1.79 ± 0.03a	1.99 ± 0.04a	2.01 ± 0.13a	2.05 ± 0.07a
Carbendazim	0.25 ± 0.10a	0.18 ± 0.02a	0.32 ± 0.11a	0.17 ± 0.04a	0.17 ± 0.02a	0.26 ± 0.11a	0.20 ± 0.08a

a All amended CZA media were prepared with indicated sources. Means followed by different lowercase letters in each line are significantly different (Tukey’s HSD, *P* < 0.05).

### Functions of CDAs in the asexual development and conidial tolerance of fungi.

**FIG 2:**
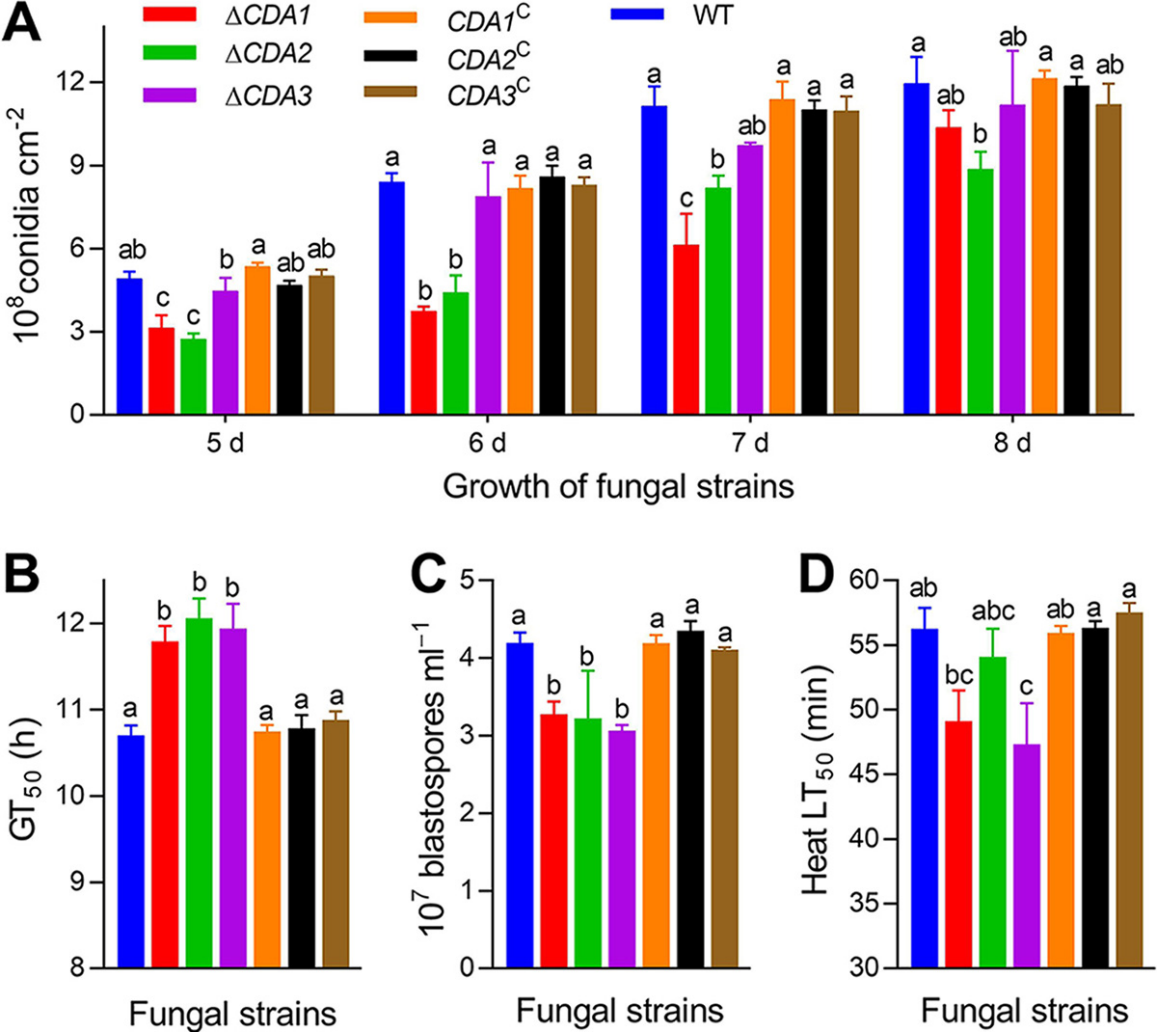
Analysis of asexual development and conidial activity of fungi. (A) Conidial yield was measured daily on SDAY from days 5 to 8. (B) The germination rate of conidia at 25°C is expressed as the GT_50_ (h). (C) Conidial production was quantified from NLB after 3 days of culture. (D) Conidial heat tolerance (LT_50_) was assessed by treating the conidia at 45°C. Different lowercase letters indicate significant differences (Tukey's HSD, *P* < 0.05). Error bars: standard deviation (SD) of 3 biological replicates.

Deletion of the three CDA genes had different effects on conidial yield. On days 5 to 7, the conidial yield of Δ*CDA1* decreased by 36.04%, 55.46%, and 44.92% compared with that of WT. Nevertheless, on day 8, the conidia production of Δ*CDA1* showed no difference from that of the control strains (Fig. 2A, *P* > 0.05). These results suggest that CDA1 may affect the early formation of conidia. Conidial production of Δ*CDA2* on days 5 to 8 decreased 25.81% to 47.68% compared with that of WT. On days 5 to 8, the conidial production of the Δ*CDA3* and the control strains was similar (Fig. 2A, *P* > 0.05). The median germination time (GT_50_) was used to assess the conidial germination ability. Our data showed that the conidial germination of the three mutants was slow, and the GT_50_ values of Δ*CDA1*, Δ*CDA2*, and Δ*CDA3* were 10.16%, 12.66%, and 11.53% later than the GT_50_ of WT, respectively (Fig. 2B).

Blastospores are an important asexual reproduction form of B. bassiana. After invading the hemolymph of the host, the fungus reproduces as yeast-like blastospores (30). The blastospore production of Δ*CDA1*, Δ*CDA2*, and Δ*CDA3* was 20.23%, 23.21%, and 27% lower, respectively, than that of WT on the third day (Fig. 2C, *P* < 0.05). Δ*CDA1* and Δ*CDA2* showed no difference in conidial heat resistance (Fig. 2D, *P* > 0.05), while Δ*CDA3* showed reduced thermotolerance. The LT_50_ of Δ*CDA3* was reduced by 15.85% compared with that of WT (56.26 ± 1.62 min) (Fig. 2D, *P* < 0.05). However, the growth features of all the gene deletion mutants were similar to those of the control strains on the chemical stress media (H_2_O_2_, CR, CAR, NaCl, MND, SDS, carbendazim, and sorbitol) that simulated the different environmental stresses (Table 1, *P* > 0.05).

### Roles of CDAs in virulence.

**FIG 3:**
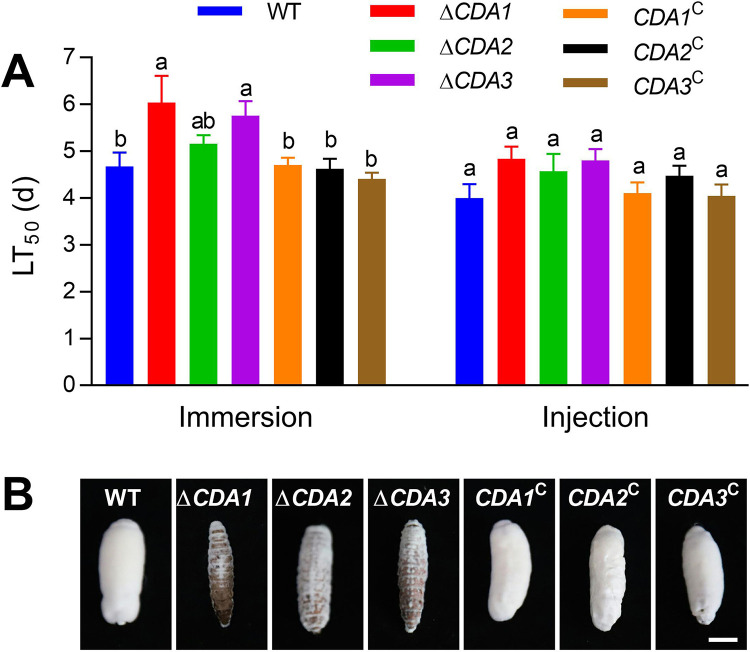
Determination of fungal virulence to larval G. mellonella. (A) The LT_50_ after cuticle infection of G. mellonella was achieved with 10^7^ conidia/mL and hemocoel infection was achieved by injection of ~500 conidia. (B) The images show the mycelia growing on the corpse surface 5 days after larval death. Different lowercase letters indicate significant differences (Tukey's HSD, *P* < 0.05). Error bars: SD of 3 biological replicates.

To investigate whether CDA gene disruption affects virulence, conidia were employed to kill Galleria mellonella larvae by cuticle infection or hemocoel infection. The LT_50_ value (days) was used to assess virulence (Fig. 3A). For cuticle infection, the mean (± SD) LT_50_ of Δ*CDA1* and Δ*CDA3* were 6.04 ± 0.57 days and 5.76 ± 0.55 days, respectively, or 29.33% and 23.34% longer than that of WT (4.67 ± 0.30 days) (Fig. 3A, *P* < 0.05). All the gene disruption strains showed results similar to those of the control strains in the hemocoel infection pathway (Fig. 3A, *P* > 0.05). In addition, a large amount of mycelia was observed on the surface of G. mellonella killed by the WT and complementation strains, while the surface of corpses killed by *CDA* deletion mutants were mostly exposed, especially those killed by Δ*CDA1* and Δ*CDA3* (Fig. 3B).

**FIG 4:**
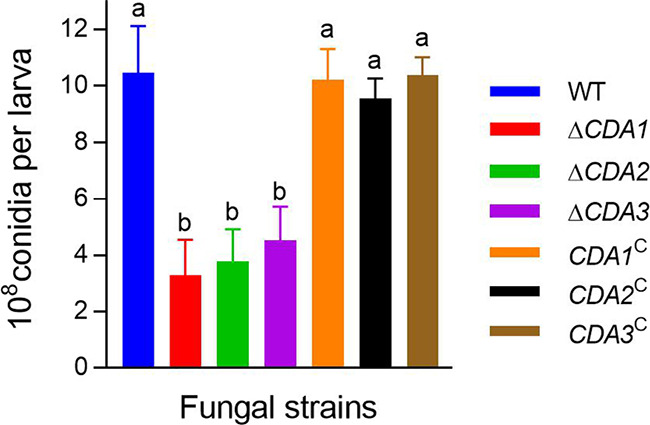
Analysis of secondary sporulation ability. Quantification conidia regenerated on the surface of dead larvae after 10 days. Different lowercase letters indicate significant differences (Tukey's HSD, *P* < 0.05). Error bars: SD of 3 repeated assays.

To further explore the secondary sporulation ability of the mutants, the corpses were cultured for 10 days. Quantitative measurements of conidiation (10^8^/conidia per larva) on the corpse surface showed that Δ*CDA1*, Δ*CDA2*, and Δ*CDA3* were 3.31 ± 1.24, 3.79 ± 1.13, and 4.54 ± 2.05, respectively, or 68.45%, 63.84%, and 56.65% lower than the WT (10.48 ± 1.65) (Fig. 4, *P* < 0.05). These data revealed that disruption of the CDA genes affects parasitism and saprophytic growth of insect pathogenic fungi.

### Roles of CDAs on chitin metabolism.

**FIG 5:**
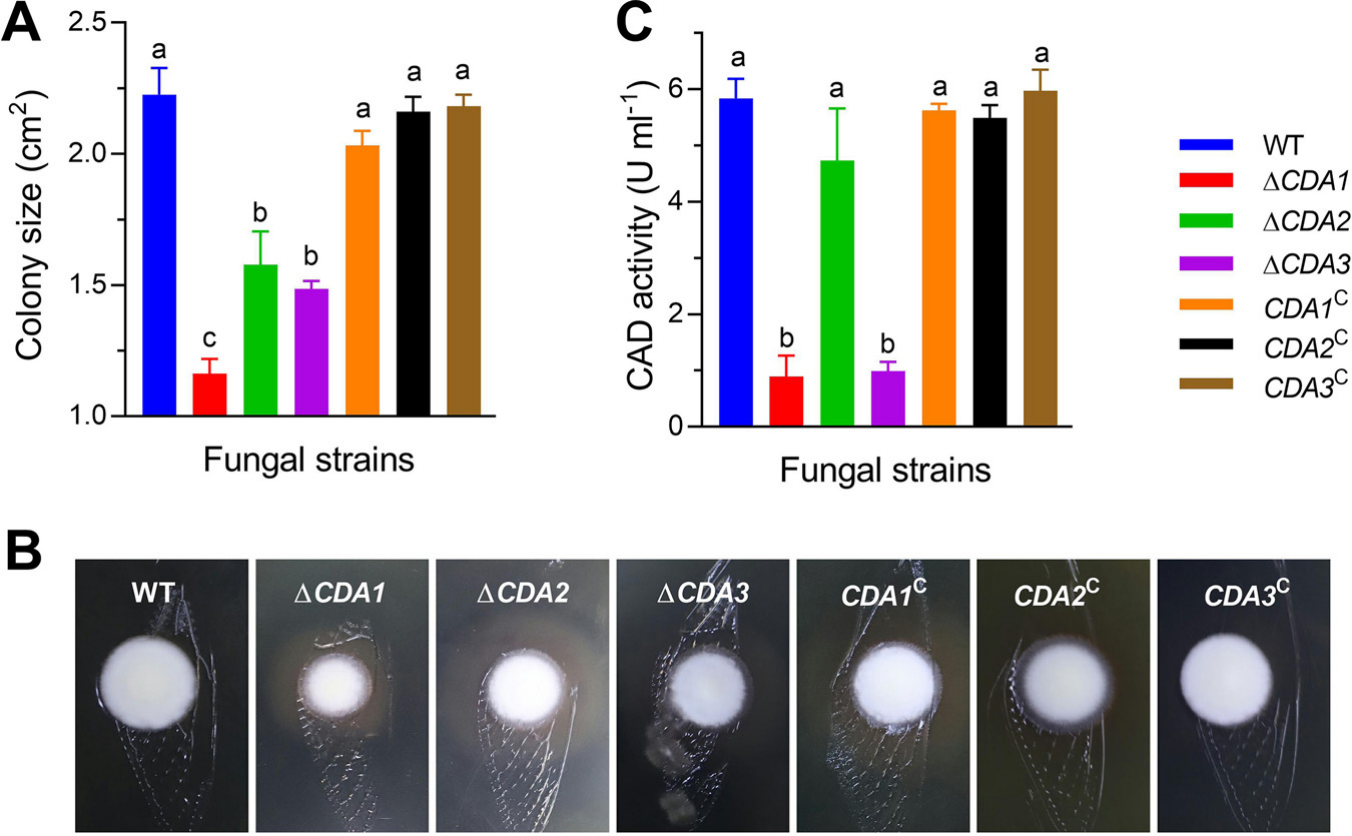
Detection of chitin deacetylation and penetration ability of fungi. (A, B) Colony size was measured and the colony growth state on medium containing cicada wings was photographed after 6 days. (C) Enzyme activity was measured in the fermentation broth of cicada decidua culture medium after 72 h of induction. Different lowercase letters indicate significant differences (Tukey's HSD, *P* < 0.05). Error bars: SD of 3 biological replicates.

Cicada wings were used to simulate the insect epidermis to verify the penetration process of the CDA mutants. All the tested strains successfully passed through the cicada wings and grew on the SDAY medium below the wings. However, after 3 days, the colony sizes of Δ*CDA1*, Δ*CDA2*, and Δ*CDA3* were 47.76%, 29.12%, and 33.29% smaller, respectively, than that of WT (Fig. 5A and B, *P* < 0.05). These results indicate that the CDA mutants had reduced penetration ability compared to WT.

To further explore the impact of CDA gene disruption on the metabolism of chitin, the deacetylation activity of the fungi was determined. Three days of culture in fermentation broth containing cicada decidua showed that the enzyme activity of Δ*CDA1* and Δ*CDA3* decreased was 84.76% and 83.04% lower, respectively, than that of WT (Fig. 5C, *P* < 0.05), indicating that CDA1 and CDA3 may play a prominent role in chitin deacetylation.

## DISCUSSION

Genes of the *CDA* family are widespread in fungi (31), for example, there are six CDA genes in T. atroviride, seven in A. fumigatus, and 10 in M. oryzae (17, 22, 32). Seven *CDAs* of Ustilago maydis have both specific and redundant functions in fungal virulence and cell wall integrity (33). In this study, three CDA genes were found to affect the yields of conidia and blastospores, cuticle infection virulence and chitin deacetylation activity of *B.* bassiana, affecting the biocontrol potential of this fungus, as discussed below.

In *Colletotrichum lindemuthianum*, ClCDA is a representative member of the CE-4 (carbohydrate esterase 4) family with many conserved amino acid motifs, including a conserved His-His-Asp zinc-binding triad (34). Most CE-4 family members are metal-dependent hydrolases, and the most common metal cations are Zn^2+^ and Co^2+^ (29, 34, 35). Structural analysis showed that the CDAs contain a catalytic metal ion, except CDA3 in B. bassiana. Similarly, for CDA from A. nidulans, no metals were added to the crystal structure, but the 2F_o_-F_c_ map showed a peak refined as Co^2+^ bound to the His-His-Asp triad (35). Although the three B. bassiana CDAs shared high homology with a template protein (GO:0005975) involved in carbohydrate metabolism, our data showed that only Δ*CDA1* exhibited slow growth on media containing no carbon source or chitin as a single carbon source.

The process of B. bassiana infection involves a series of morphological transformations (3, 30, 36). Chitosan has better flexibility and solubility than chitin, and deacetylation can increase cell wall plasticity (6). Our data showed a decrease in conidial production on days 5 to 7 for Δ*CDA1* and Δ*CDA2*. The three gene deletion mutants showed slower germination and reduced blastospore production. Chitin deacetylation occurs in response to physical stimuli during the early stages of germination morphogenesis (22), and environmental stimuli can trigger fungal cell wall remodeling, including the rearrangement of chitin and chitosan (37, 38). The presence of the CDA gene is important for the correct formation and rigidity of S. cerevisiae ascospores. The second layer of the ascospore wall consists of chitosan. Acetylation may disrupt the interactions between the chitosan layer and the outermost macromolecule containing dimerylysine, resulting in a loose spore wall structure that is susceptible to hydrolase and stress conditions (13, 14). Although the production of conidia, as well as their vitality, decreased, all the strains showed no difference in growth parameters under the various stress conditions tested, except for CDA3, which showed a difference under heat stress. Similarly, when grown in medium containing the cell wall inhibitor CR, CFW, or SDS, the Pyricularia oryzae CDA mutant Δ*Pocda7* showed no difference from the wild type under the same conditions (39). The cell wall integrity of the CDA gene deletion mutants may be maintained by upregulation of other components in the cell wall (22).

Some cuticle-degrading enzymes are virulence determinants (30). The association between CDA activity and virulence has been revealed in pathogenic fungi. In C. neoformans, CDA1 and its activities are essential for fungal virulence. When the CDA1 is absent, the mutants have less chitosan content or a lower degree of acetylation, which makes them more susceptible to the host chitosanase (20). Ecp6, the effector protein of the plant pathogen *Cladosporium fulvum*, may possess CDA activity and participate in chitin binding. During infection, the cell wall-released chitin oligosaccharides are sequestered by Ecp6 to prevent the induction of host immunity (40, 41). Partial deacetylation of exposed chitin polymers or elicitor active chitin oligomers is considered to be a mechanism for escaping the host immune response (42). *M. anisopliae* CDA may not only modify the cuticular chitins of insects or the self-cell wall to facilitate penetration but also alter the cell wall to defend against insect chitinase (26). In this study, Δ*CDA1* and Δ*CDA3* showed decreased virulence during surface infection, which was consistent with the downregulation of CDA enzyme activity and weaker penetrability observed (Fig. 5A and B). Unlike cuticle infection, hemocoel injection does not require penetration of the insect cuticle, and in the virulence test, the deletion mutants had results similar to those of the control strains. In addition, the germination ability and blastospore yield of the gene deletion mutants were decreased, which may also be one of the important reasons for the decline in fungal virulence. The mechanical stress generated during germination can affect the penetration process. Blastospores have a large surface area to volume ratio, which makes it easier for the fungus to absorb nutrients. Until the nutrients are depleted, the blastospores convert into hyphae. The mycelia growing *in vitro* produce conidia and start a new infection process (30). In addition, deletion of the three genes led to a great decrease in fungal secondary spore production. The lack of CDA not only affected fungal parasitism but also affected the saprophytic life of the fungi.

## MATERIALS AND METHODS

### Source of strains and raise conditions.

In this study, the R. W. Holley Center for Agriculture and Health (Ithaca, NY, USA) provided the wild-type strain *B.* bassiana ARSEF 2860 (WT). Both the WT and mutant strains were incubated on Sabouraud dextrose agar media at 25°C (SDAY: 4% glucose, 1% peptone, 1% yeast paste, and 1.5% agar). Depending on the plasmid selection marker used, appropriate antibiotics were added to Luria-Bertani (LB: 1% peptone, 1% NaCl, 0.5% yeast extract, and 1.5% agar). The transformation strain, Agrobacterium tumefaciens AGL-1, was incubated in yeast extract broth (YEB: 0.5% sucrose, 0.1% yeast extract, add 1% peptone and 0.05% MgSO_4_).

### Phylogenetic tree, structure, and transcriptional analysis of CDAs.

Using the S. cerevisiae CDA protein sequences CDA1 (NP_013410.1) and CDA2 (NP_013411.1) as queries, the B. bassiana genome was searched by basic local alignment search tool for proteins (BLASTp) from the National Center for Biotechnology Information (NCBI) (43). The ClustalW algorithm was used for sequence alignment (44), and MEGA-X software (https://www.megasoftware.net/) was used to perform the phylogenetic analysis with the neighbor-joining method (1,000 bootstrap replicates). CDA structural models predictions and consensus prediction of Gene Ontology (GO) terms were performed by using the Iterative Threading Assembly Refinement (I-TASSER) server (https://seq2fun.dcmb.med.umich.edu//I-TASSER/) (45). The 3D structures of the CDAs were colored, and ligand docking was performed by using PyMoL software (46). The putative signal peptide site of CDAs were predicted by SignalP 4.1 (47).

To assess transcriptional change of CDAs over the time of WT development during the various stages and infection process, we simulated the various development stages of B. bassiana by three media of different growth conditions. For the mycelium growth medium, 100 μL conidial suspensions (a 10^7^ conidia/mL concentration was maintained unless otherwise stated) of WT were plated on SDAY for 4 days. For the medium used to simulate conidiation stage, aliquots of conidial suspension were cultured on SDAY for 7 days. One hundred μL conidial suspensions were inoculated in nitrogen-limited broth (NLB: glucose 4%, NH_4_NO_3_ 0.4%, KH_2_PO_4_ 0.3%, MgSO_4_ 0.3%) medium for 4 days to simulate the stage of blastospore production of fungi. For the fungal infection process, the larvae of G. mellonella were divided into three groups with 10 larvae in each group and immersed in 30 mL conidial suspension for 10 s, and the larvae were reared in petri dishes at 25°C for 3 days. Larvae from each group were ground in liquid nitrogen every other day for RNA extraction. Total RNA were extracted and reversely transcribed into cDNA using the previously described method (48, 49). Each cDNA sample (10-fold dilution) was used as the template to quantify CDAs transcript through qRT-PCR with the primers listed in Table S1. The fungal 18S rRNA was used as internal standard. Relative transcript levels were calculated as the transcript ratios of CDAs of each stage versus the stage of mycelium growth or infection process of each day versus first day using the 2^-ΔΔCT^ method (48).

### Disruption and complementation of the CDA genes.

CDA gene mutants were constructed by homologous recombination as described previously (50, 51). The specific fragment was cloned from the genome of the WT strain and was integrated into the appropriate position of E. coli (Escherichia coli) P0380 by using the corresponding endonucleases and ligases to generate gene deletion and complementation vectors. Table S1 shows the primers used for the DNA amplification process. The constructed vectors were transformed by Agrobacterium-mediated transformation to generate gene deletion strains and complementation strains (52, 53). Phosphinothricin (200 μg/mL) was used to screen the putative deletion mutants based on *bar* resistance, and the complemented mutants were identified by screening the for resistance to 10 μg/mL chlorimuron ethyl (27, 54). PCR and qRT-PCR were used to verify correct recombination events, and the primers used are listed in Table S1.

### Analysis of asexual growth.

To measure the ability of the strains to produce conidia, 200 μL conidial suspensions were uniformly coated on SDAY plates covered with cellophane. Beginning on day 5, three mycelial plugs with a diameter of 5 mm were punched from each plate every other day. The mycelial plugs were crushed and suspended in 1 mL of 0.02% Tween 80, and the cells were counted with a hemocytometer.

To evaluate the conidial germination ability, 100 μL conidial suspensions were inoculated in 900 μL of germination broth (GB: peptone 0.5%, sucrose 2%). The germination solution was oscillated at 180 rpm and 25°C, and the ratio of spore germination was calculated by using a hemocytometer every 2 h until all the fungal conidia had germinated. The 50% conidial germination activity was measured by determining the GT_50_ (h) of the conidia, and the results were calculated according to the germination trend of fungal conidia.

To evaluate the blastospore production of the strains, 200 μL conidial suspensions were added to 100 mL of NLB medium for shaking culture at 25°C. On day 3, the blastospore yield (cells/mL) was measured.

### Assays of fungal growth in various nutrient and stress conditions.

To verify the growth state of the gene-deleted and control strains (WT and complemented mutants) on different media, 1 μL conidial suspensions were spotted on the plate according to a previously described method (55). The media included SDAY, 1/4 SDAY (1/4 of each nutrient in SDAY), Czapek-Dox Agar (CZA: sucrose 3%, NaNO_3_ 0.3%, K_2_HPO_4_ 0.1%, KCl 0.05%, MgSO_4_ 0.05%, and FeSO_4_ 0.001%; add 2% agar) and CZAs modified with several carbon or nitrogen sources or without a C source (CZA-C), N source (CZA-N), or both (CZA-CN). In the modified CZAs, the CZA carbon source was replaced with GlcNAc (CZA-GlcNAc) or colloidal chitin (CZA-chitin) (28).

To test the stress tolerance of the strains, 1 μL conidial suspensions were placed on CZA plates, and NaCl (30 μg/mL), CR (30 μg/mL), CAR (0.2 μg/mL), sorbitol (182.17 mg/mL), MND (0.2 nM), and 0.02% SDS and H_2_O_2_ (2 mM) were used as chemical stress sources. The size of the colonies on several plates was assessed after 7 days of culture at 25°C. The growth rate index was calculated from the colony area (cm^2^) of all the strains.

To evaluate the heat resistance of the conidia, conidia samples were cultured at 25°C for 24 h after treatment at 45°C for 0 to 90 min. To quantify conidial thermotolerance, the median lethal time (LT_50_, min) was used to measure the conidial germination rate.

### Virulence evaluation.

Fungal virulence tests were carried out on the G. mellonella larvae (~400 mg per larva). For cuticle infection and hemocoel infection, 30 larvae were immersed in a 30-mL suspension of conidia for 10 s or injected with a 1 μL 5 × 10^5^ conidial (mL^−1^) suspension. Equal doses of 0.02% Tween 80 were used as the blank control for both treatments. All larvae were cultured in 12 h light and 12 h darkness at 25°C, and the number of larval deaths was recorded every 12 h. As an indicator to assess virulence, the mortality trend index LT_50_ (days) was obtained by probability analysis. After the larvae died, the corpses were incubated at 25°C under saturated humidity. The development of hyphae on the insect surface was photographed after 5 days.

To evaluate the ability of the fungi to produce conidia, dead larvae were incubated for 10 days. The corpses were strongly agitated in 0.02% Tween 80 for 10 min, and the conidia from each larva was measured by a hemocytometer.

To explore whether deletion of *CDA* genes affects the metabolism of chitin during the fungal penetration process, the insect cuticle was simulated by using the cicada (*Cryptotympana atrata*) wings. A total of 1 μL of a 1 × 10^6^ conidia/mL conidial suspension was applied to the aseptically treated wings placed on SDAY. The wings were removed after 3 days of incubation, and the remaining medium was further incubated at 25°C for 3 days (28, 56).

### Assay for CDA activity.

The activity of CDA was determined by a modified method (57). For this, 100-μL conidial suspensions of the deletion strains and the control strains were inoculated in 100 mL of modified medium (glucose 0.5%, yeast extract 0.5% [NH_4_]_2_SO_4_ 0.4%, KH_2_PO_4_ 0.15%) (58) and cultured at 25°C for 3 days. Then, fungal cells were collected and inoculated into 100 mL of CZB-CN (agar-free CZA-CN) supplemented with 1% cicadae. The media were incubated at 25°C for 3 days. The enzymatic reaction was based on a previously described method (59).

### Statistical analysis.

One-way ANOVA and Tukey's honest significant difference (HSD) test were used to distinguish the differences and the mean values of each treatment phenotype, respectively.
