# Supplementary material for: Three Chitin Deacetylase Family Members of Beauveria bassiana Modulate Asexual Reproduction and Virulence of Fungi by Mediating Chitin Metabolism and Affect Fungal Parasitism and Saprophytic Life
Source: Microbiol Spectr. 2023 Feb 14;11(2):e04748-22. doi: 10.1128/spectrum.04748-22 (PMC10101055; doi:10.1128/spectrum.04748-22)
Supplement: Supplemental file 1 — Supplemental material. Download spectrum.04748-22-s0001.pdf, PDF file, 1.6 MB [file spectrum.04748-22-s0001.pdf]

Supplemental Material FOR Publication

Three chitin deacetylase family members of *Beauveria bassiana* modulate asexual reproduction and virulence of fungi by mediating chitin metabolism and affect fungal parasitism and saprophytic life

Jia-Hua Liu[1], Jing-Chong Dong[1], Jun-Jie Gao[1], Xin-Peng Li[1], Shun-Juan Hu[1], Juan Li[2], Wen-Xiao Hu[1], Xian-Yan Zhao[1], Juan-Juan Wang[2], Lei Qiu[1*]

[1] *State Key Laboratory of Biobased Material and Green Papermaking, Qilu University of Technology, Shandong Academy of Sciences, Jinan, China*

[2] *School of Biological Science and Technology, University of Jinan, Jinan, China*

[*]Correspondence author

Lei Qiu*: State Key Laboratory of Biobased Material and Green Papermaking, Qilu University of Technology, Shandong Academy of Sciences, Jinan, Shandong 250353, PR China. E-mail: qiulei.2005@163.com*

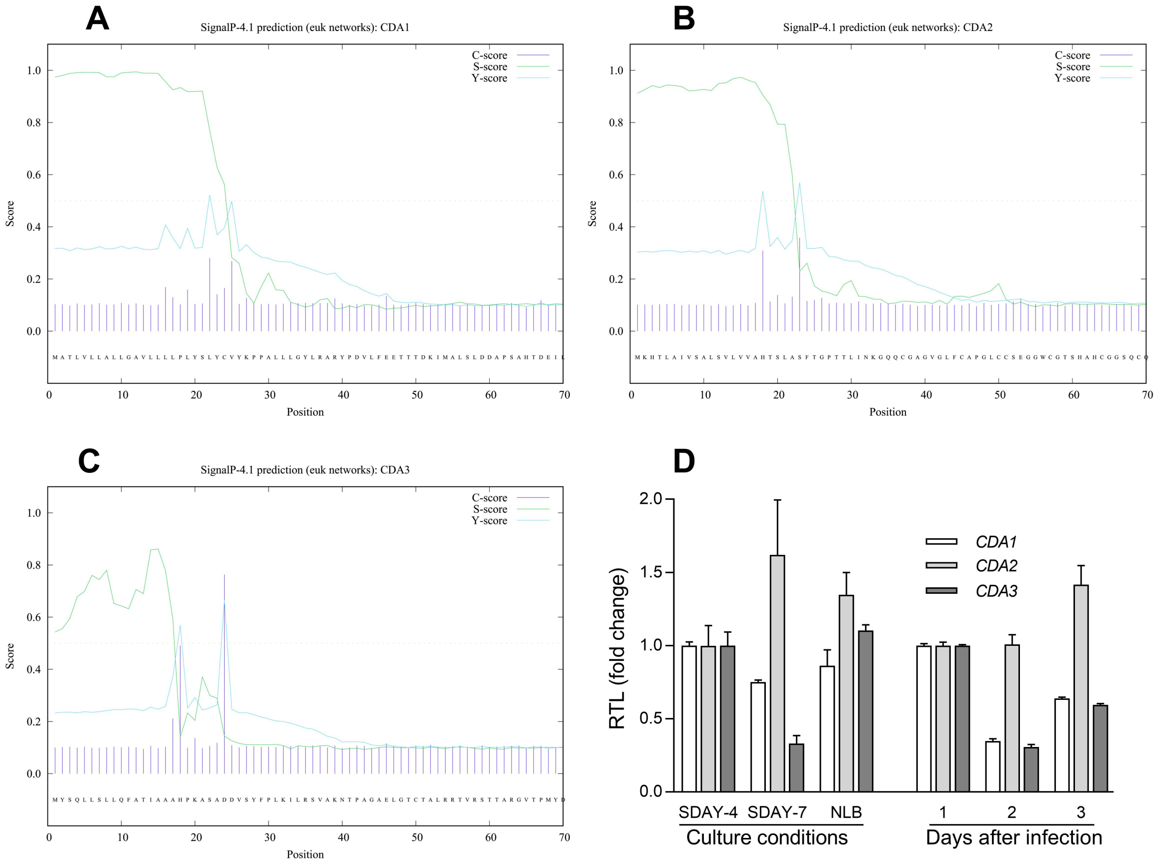

FIG S1 Expression features of CDAs of *B. bassiana*. (A-C) SignalP 4.1 server prediction plots for the signal peptides and the cleavage site of CDA1, CDA2 and CDA3(A-C). C-score: cleavage site value, S-score: signal peptide value, Y-score: geometric average between the C-score. The cleavage site is predicted to be at the position of the maximum value in Y-score. (D) Relative transcript levels (RTL) of *CDAs* genes during various developmental stages and infection process of WT. The stages of the mycelium growth, conidiation and blastospore production were simulated by culturing *B. bassiana* on SDAY plate for 4 days, on SDAY plate for 7 days and in NLB for 4 days, respectively. The transcriptional levels of mycelium growth stage and the first day of the infection process were used as standards. Error bars: SD from three cDNA samples per treatment.

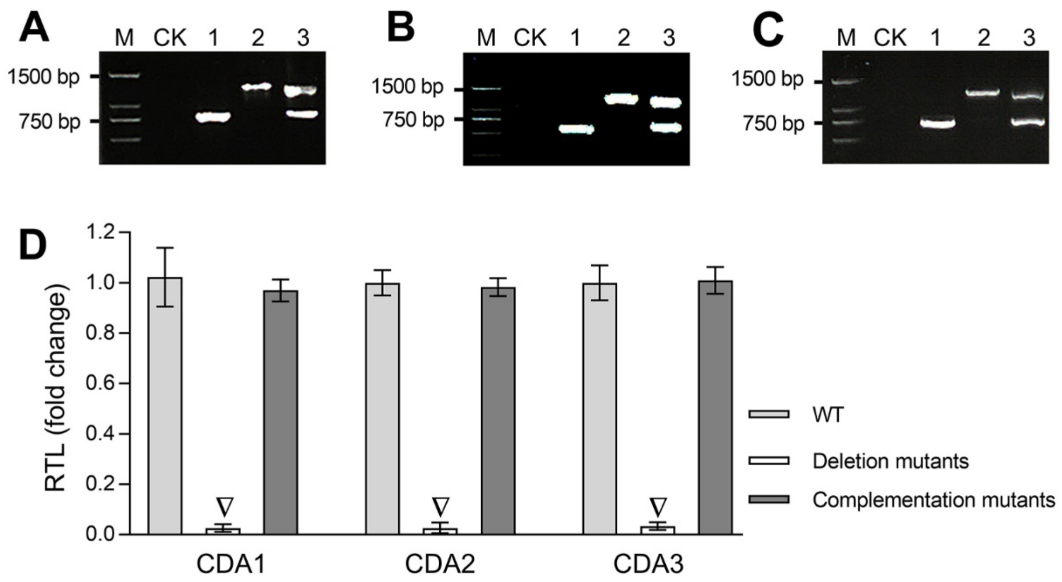

FIG S2 The CDA1, CDA2 and CDA3 mutants were identified by PCR and qRT-PCR. (A-C) CDA1, CDA2 and CDA3 were successfully deleted and complemented via PCR verification. Lane 1: DNA Marker. Lane 2: Control Check. Lane 3: WT. Lane 4: Δ*CDA1*(A), Δ*CDA2*(B) and Δ*CDA3*(C). Lane 5: *CDA1*^{C}(A), *CDA2*^{C}(B) and *CDA3*^{C}(C). (D) Identification of *CDAs* deletion by qRT-PCR with the primer pairs listed in Table S1.

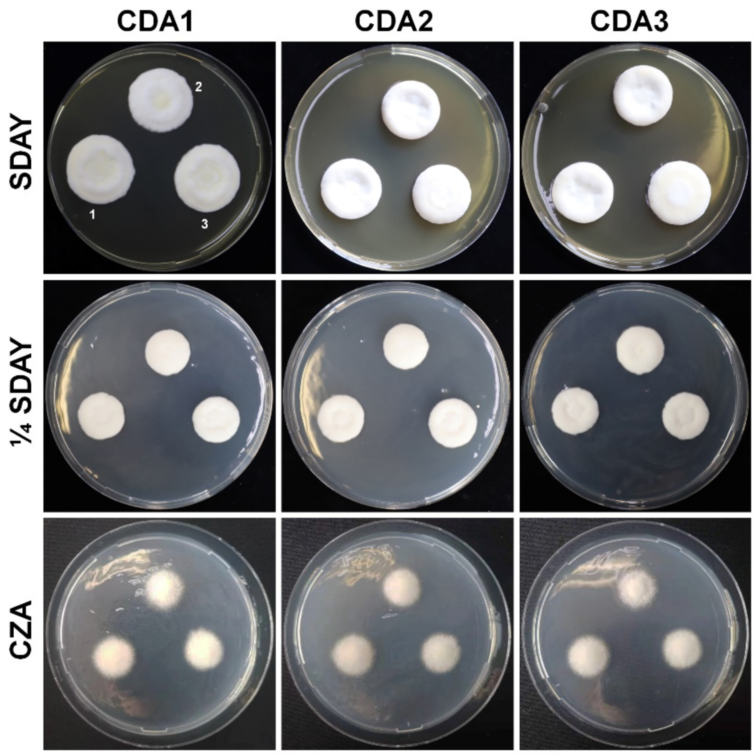

FIG S3 Images of colonies grown on SDAY, 1/4 SDAY and CZA plates. Labels on plate are as follows: 1 = WT. 2 = gene deleted mutants. 3 = complementation strains. The same sequence of samples was used in all plates.

Table S1. Paired primers designed for manipulation of CDAs in *B. bassiana*.

Primers	Paired sequences (5′-3′)*	Purpose
CDA1up-F/R	AAAGAATTCGCCGCACGAGGAGAAGA/AAAGGATCCCGCAAGACGCCGAGAAT	Cloning 5′ *CDA1*
CDA1dn-F/R	AAAAGATCTGAGGCGTTGGGAATAGACA/AAAACTAGTATCCAGCGACGGAAGAG	Cloning 3′ *CDA1*
CDA1fl-F/R	GGGGACAAGTTTGTACAAAAAAGCAGGCTAATGCTTTGAGCGAGAACC/ GGGGACCACTTTGTACAAGAAAGCTGGGTTCATCTAGGGCTAAGGAGTATCT	Cloning full-length *CDA1*
pCDA1-F/R	AAAAGACCACCACGGACAAGA/AAATCCCAATCCACGAGCAA	PCR detecting *CDA1*
qCDA1-F/R	TTTTCGTCATTGGCGCACAG/ATGGTTCCACCGACTTGACC	qRT-PCR detecting *CDA1*
CDA2up-F/R	AAAGAATTCGCAGCGGAAAGTCGTTGTT/AAAGGATCCGGTTCCAATCGCAAATCCTAG	Cloning 5′ *CDA2*
CDA2dn-F/R	AAACTCGAGGGCCCTACTACCCTAATAAACA/AAAACTAGTGCGGCATAACGCCCTCT	Cloning 3′ *CDA2*
CDA2fl-F/R	GGGGACAAGTTTGTACAAAAAAGCAGGCTGTATTTGTGGCACTGCTTCC/ GGGGACCACTTTGTACAAGAAAGCTGGGTGCTTGGCTTCTTCTTGTTGTA	Cloning full-length *CDA2*
pCDA2-F/R	AAA ACCTCGGGCAACCAGTC/AAAGTTCCGCACCATCCTCC	PCR detecting *CDA2*
qCDA2-F/R	GCTCTCAGTGCCAACTGGAT/AAGCGTTCCTTGGAGAGACG	qRT-PCR detecting *CDA2*
CDA3up-F/R	AAAGAATTCTGTCCTTTCGGAGGTTTG/AAAGGATCCTCGCCTTTCAGTGATGGTA	Cloning 5′ *CDA3*
CDA3dn-F/R	AAACTCGAG GGAGTTGGGCACCTGTA/AAATCTAGATGGCTCTGCGATTCATAC	Cloning 3′ *CDA3*
CDA3fl-F/R	GGGGACAAGTTTGTACAAAAAAGCAGGCTCAAGCACCACGACAAGAAT/ GGGGACCACTTTGTACAAGAAAGCTGGGTCATCAACAATCCGAAATCC	Cloning full-length *CDA3*
pCDA3-F/R	AAAGCACAACAATGCGTGAACT/AAACATCGCCAAGAGGATAAGC	PCR detecting *CDA3*
qCDA3-F/R	GCATTCTTGGGGTGGTCTCA/TCATGCTCAATCTGCAGCCA	qRT-PCR detecting *CDA3*

* Underlined regions denote the sites of restriction enzyme in the *CDA1* deletion mutant (*Eco*RI / *Bam*HI and *Bgl*II / *Spe*I), *CDA2* deletion mutant (*Eco*RI / *Bam*HI and *Xho*I / *Spe*I), *CDA3* deletion mutant (*Eco*RI / *Bam*HI and *Xho*I / *Xba*I) or gateway fragments exchanged for the targeted *CDA1, CDA2* and *CDA3* complementation mutants.
